# One-Stage Transcatheter Aortic Valve Implantation and Transcatheter Edge-to-Edge Tricuspid Valve Repair for Double Valve Dysfunction in a High-Risk Patient

**DOI:** 10.7759/cureus.45648

**Published:** 2023-09-20

**Authors:** Besart Cuko, Massimo Baudo, Julien Ternacle, Lionel Leroux, Thomas Modine

**Affiliations:** 1 Department of Cardiology and Cardiovascular Surgery, Hopital Cardiologique de Haut-Leveque, Bordeaux, FRA; 2 Cardiac Surgery, ASST Spedali Civili di Brescia, University of Brescia, Brescia, ITA; 3 Department of Cardiology and Cardiovascular Surgery, Hopital Cardiologique de Haut-Leveque, Bordeaux University Hospital, Bordeaux, FRA

**Keywords:** transcatheter aortic valve implantation (tavi), one stage, tricuspid edge-to-edge valve repair, aortic valve stenosis, tricuspid valve regurgitation, tricuspid valve repair, aortic valve disease

## Abstract

A 75-year-old female patient was referred to our institution for severe symptomatic low-flow low-gradient aortic valve stenosis and tricuspid valve regurgitation (TR) associated with heart failure. After multidisciplinary discussion, the patient was scheduled for one-stage totally percutaneous treatment of her valve lesions by transcatheter aortic valve implantation (TAVI) and transcatheter edge-to-edge tricuspid valve repair (TEER) through transfemoral access. The patient had an uneventful hospital stay and was discharged home on the third postoperative day. During the following 24 months, the patient did well with regression of her heart failure signs and symptoms.

## Introduction

Severe tricuspid valve regurgitation (TR) may coexist in patients with severe aortic valve stenosis with an increase in morbidity and mortality [1]. As shown in the current 2021 European Society of Cardiology (ESC)/European Association for Cardio-Thoracic Surgery (EACTS) guidelines for the management of valvular heart diseases, concomitant tricuspid valve surgery in patients undergoing surgical aortic valve replacement is recommended (recommendation class 1, evidence level C), but there are no clear indications on the management of TR in patients undergoing transcatheter aortic valve implantation (TAVI) [2]. We present a case report of the one-stage procedure of percutaneous TAVI and transcatheter edge-to-edge tricuspid valve repair (TEER) in a high-risk patient suffering from severe symptomatic low-flow low-gradient aortic valve stenosis and TR with heart failure signs.

## Case presentation

**Figure 1:**
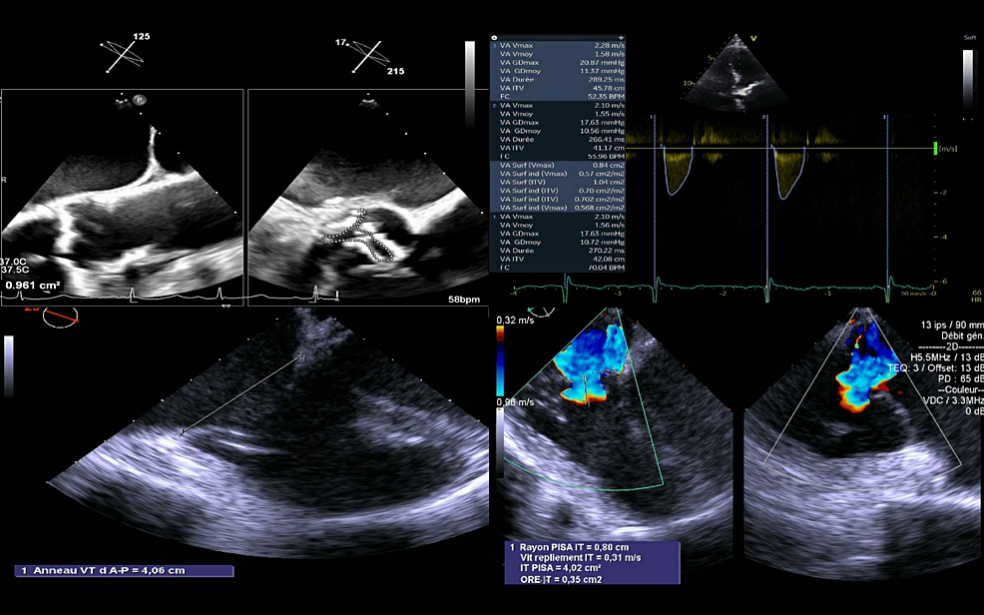
Preoperative echocardiogram images showing severe AS and severe TR AS: aortic valve stenosis; TR: tricuspid valve regurgitation

A 75-year-old female patient was referred to our institution for signs of heart failure and dyspnea in the New York Heart Association (NYHA) functional classes III-IV. Her cardiovascular history included arterial hypertension, dyslipidemia, permanent atrial fibrillation in treatments with non-vitamin K oral anticoagulation and previous multiple radiofrequency transcatheter ablations, and ischemic cardiomyopathy with coronary artery disease in medical treatment. Furthermore, she had associated Meniere’s disease, osteoporosis with osteoporotic multiple vertebral fractures, and breast cancer surgically treated with adjuvant radiotherapy and chemotherapy. Transthoracic and transesophageal echocardiograms showed severe low-flow low-gradient aortic valve stenosis (LFLG-AS) with heavily calcified leaflets (valve area: 1 cm^2^; left ventricular ejection fraction: 65%) and severe functional TR due to tricuspid annular dilatation (antero-posterior diameter: 40 mm) with a systolic pulmonary arterial pressure of 55 mmHg (Figure 1). 

The multidisciplinary Heart Team conducted a comprehensive evaluation and deliberated on potential treatment choices. Their assessment indicated a notably elevated risk of irreversible health complications or deaths linked to traditional surgeries. As a result, it was determined that a fully percutaneous transcatheter approach would be the most suitable and optimal treatment option for addressing the LFLG-AS and TR. Patient’s informed consent for the procedure and data collection for research purposes was obtained.

**Figure 2:**
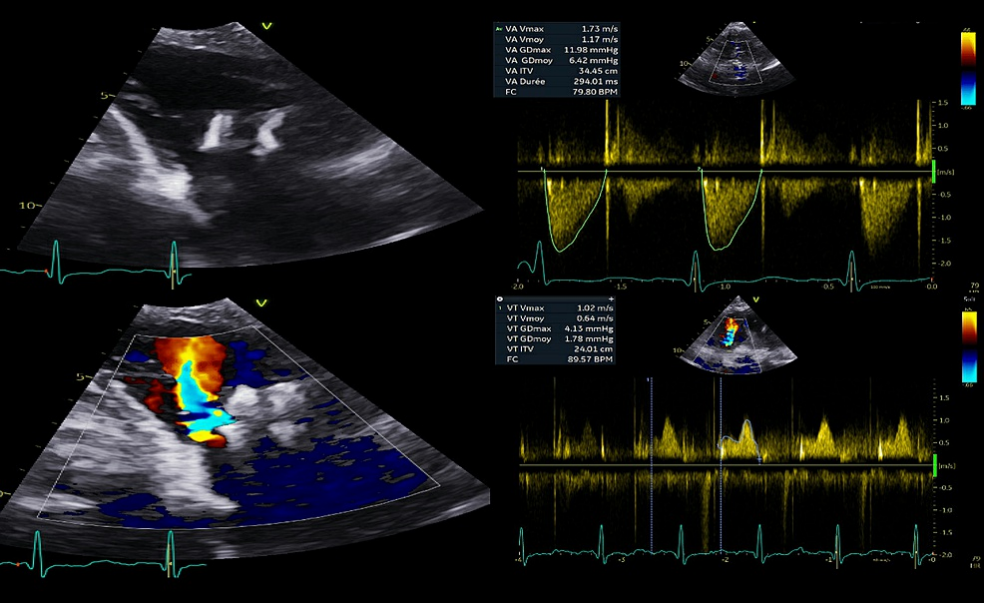
Postprocedural echocardiogram showing a well-functioning TAVI and TEER TAVI: transcatheter aortic valve implantation; TEER: transcatheter edge-to-edge tricuspid valve repair

The procedure was performed according to the standard institutional TAVI and TEER protocol in a hybrid operating room under general anesthesia. Intravenous unfractionated heparin was given intraoperatively to achieve an activated clotting time (ACT) greater than 250 seconds. First, through the right femoral artery, the TAVI delivery system was advanced across the calcified aortic valve. Under transesophageal and fluoroscopic guidance, a 26 mm Edwards Sapien 3 (Edwards Lifescience, Irvine, CA, USA) valve was implanted. Subsequently, through the right femoral vein, the Pascal delivery system (Edwards Lifescience, Irvine, CA, USA) was advanced till the tricuspid valve, and two clips were deployed to grasp the anterior and septal leaflets. Then, percutaneous access hemostasis was achieved using a pre-closure technique with the suture-mediated ProGlide device. The total procedure time was 150 min. At the end of the procedure, the mean aortic valve gradient was 5 mmHg with a valve area of 2.2 cm^2^ and the TR was reduced to mild grade. There was no change between the pre- and post-procedural electrocardiogram. Post-procedural recovery was uneventful with a good hemodynamic response. The patient was discharged home three days later. Pre-discharge transthoracic echocardiogram showed a good result of transcatheter aortic valve implantation and tricuspid valve repair (Figure 2). 

At the 24-month follow-up, the patient was in good clinical conditions (NYHA class I). Transthoracic echocardiography revealed a well-functioning TAVI without anomalous paraprosthetic leaks and a stable mild tricuspid residual regurgitation without an altered left or right ventricular function.

## Discussion

Aortic stenosis (AS) is a commonly encountered and potentially serious condition. In this context, TAVI through the transfemoral approach is increasingly being adopted as the preferred treatment method for elderly individuals and/or those at high surgical risk. This approach has shown in this subset of patients a notable decrease in both the overall mortality and cardiac-related mortality rates when compared to conventional surgical aortic valve replacement [3]. Indeed, AS combined with TR is associated with high mortality rates regardless of the AS treatment [4]. Severe AS results in the left ventricular remodeling due to pressure overload, followed by right ventricular involvement and TR representing a significant risk in the overall mortality [5,6]. Nowadays, there is an increasing focus on the combination of TR and AS [1,7]. In the current landscape where TAVI is routinely performed, it is crucial to carefully evaluate the most effective treatment approaches for patients who present with both AS and TR. Especially among the elderly or those considered high risk due to advanced valvular heart conditions, employing multiple transcatheter interventions could potentially lead to improved clinical outcomes.

Ongoing research is exploring various transcatheter repair or replacement options for the tricuspid valve in cases where therapeutic choices for TR are limited [8]. In a recent multicenter study, TEER was found to be safe and effective with sustained benefits in symptomatic moderate or severe TR at two years follow-up [9]. Furthermore, in a recent meta-analysis conducted by Badwan et al., a notable achievement in procedural success was observed, accompanied by improvements in NYHA functional class [10]. To our current knowledge, there have been no reported cases of a single-step totally percutaneous aortic and tricuspid valve intervention with TAVI and TEER. Thus, the fundamental aspect of this case report lies in demonstrating the feasibility, safety, and effectiveness of this procedure as a valuable treatment option for high-risk patients. While combined open surgery remains the established standard for treating valve-related heart conditions, it is often incompatible for patients deemed at high risk.

In this era marked by rapid technological advancements in the management of transcatheter valve-related heart diseases and the execution of various transcatheter valvular procedures, it becomes crucial to consider alternative options. These alternatives should be assessed by a multidisciplinary Heart Team. The armamentarium of transcatheter valve therapies is expanding the range of treatment options available to clinicians, particularly for patients who were previously considered too high risk for surgical interventions.

## Conclusions

This case underscores the potential of combining percutaneous transcatheter interventions as an alternative treatment option for high-risk patients with complex valvular conditions. As the field of transcatheter valve therapies continues to rapidly evolve, such combined approaches hold promise in expanding the therapeutic options available to clinicians. This case report contributes to the growing body of evidence suggesting that carefully selected patients after Heart Team discussion can benefit from these innovative procedures, ultimately leading to improved clinical outcomes and enhanced quality of life.
